# Mortality in severely injured patients: nearly one of five non-survivors have been already discharged alive from ICU

**DOI:** 10.1186/s12871-020-01159-8

**Published:** 2020-09-23

**Authors:** Uwe Hamsen, Niklas Drotleff, Rolf Lefering, Julius Gerstmeyer, Thomas Armin Schildhauer, Christian Waydhas, TraumaRegister DGU

**Affiliations:** 1 Department of General and Trauma Surgery, BG University Hospital Bergmannsheil, Buerkle de la Camp Platz 1, 44789 Bochum, Germany; 2 Institute for Research in Operative Medicine (IFOM), University Witten-Herdecke, Ostheimer Str. 200, 51109 Cologne, Germany; 3 Medical Faculty University Duisburg-Essen, Essen, Germany

**Keywords:** Quality management, Normal ward, RISCII, Failure to rescue, Frailty, Risk-adjustment, Comfort care

## Abstract

**Background:**

Most trauma patients admitted to the hospital alive and die later on, decease during the initial care in the emergency department or the intensive care unit (ICU). However, a number of patients pass away after having been discharged from the ICU during the initial hospital stay. On first sight these cases could be seen as “failure to rescue” of potentially salvageable patients. A low rate of such patients might be a potential indicator of quality for trauma care on ICUs and surgical wards.

**Methods:**

Retrospective analysis of the TraumaRegister DGU® with data from 2015 to 2017. Patients that died during the initial ICU stay were compared to those who were discharged from the initial ICU stay for at least 24 h but died later on.

**Results:**

A total of 82,313 trauma patients were included in the TraumaRegister DGU®. In total, 6576 patients (8.0%) died during their hospital stay. Out of those, 5481 were admitted to the ICU alive and 972 patients (17.7%) were discharged from ICU and died later on. Those were older (mean age: 77 vs. 68 years), less severely injured (mean ISS: 23.1 vs. 30.0 points) and had a longer mean ICU length of stay (10 vs. 6 days). A limitation of life-sustaining therapy due to a documented living will was present in 46.1% of all patients who died during their initial ICU stay and in 59.9% of patients who died after discharge from their initial ICU stay.

**Conclusions:**

17.7% of all non-surviving severely injured trauma patients died within the hospital after discharge from their initial ICU treatment. Their death can partially be explained by a limitation of therapy due to a living will. In conclusion, the rate of such late deaths may partially represent patients that died of potentially avoidable or treatable complications.

## Background

Traumatic injuries are a major cause of death and disability. Worldwide, almost 10% of all deaths are related to trauma [1]. Over the last decades, improvements in trauma care in the fields of prehospital care, emergency department (ED) care, operation techniques and intensive care led to a constant reduction of mortality rates [2, 3]. Most established quality indicators focus on the initial trauma care before and during admission [4–6].

In general and elective surgery a number of studies suggested that a relevant proportion of patients decease within the hospital after discharge from their initial (post-operative) stay in the ICU. This group may represent potentially preventable deaths [7–10]. To detect and treat patients on general wards with major complications in an early stage, different strategies have been developed to lower both readmission rates to the ICU and mortality [11] Early detection of patients at risk may be achieved by use of warning scores and other systematic tools of monitoring or implementation of medical emergency teams [12, 13].

Whether these aspects of improved care for surgical patients are also relevant for the care of severely injured patients is not well known. The failure to rescue (FTR) rate was described as a more precise and therefore superior characterisation of post-operative care quality than mortality or complication rates in elective surgery [14]. It was first defined in 1992 as the mortality rate among patients with complications. However it has since been rarely applied to trauma and acute care surgery [15].

In trauma patients it can be difficult or even impossible to distinguish between preventable complications and adverse events due to “natural course of the trauma”. Thus, reliable rates of failure to rescue are still lacking [16, 17]. In order to observe and measure the quality of postoperative care, an approach could be to analyse severely injured trauma patients who have been discharged to the general ward and die after an initial treatment on an intensive care unit. The aim of this study was to determine the rate of late mortality, characteristics of patients and to compare late deaths with non-survivors who died during their initial ICU stay. We expect that this approach is a simple way to retrospectively identify a cohort of potentially preventable fatal complications, even if not regognized or defined during treatment and therefore eligible for quality management and benchmarking, especially in large trauma registries.

## Methods

### Study design and setting

The TraumaRegister DGU® of the German Trauma Society (Deutsche Gesellschaft für Unfallchirurgie, DGU) was founded in 1993. The aim of this multi-center database is a standardised and pseudo anonymised documentation of severely injured patients.

Data were collected prospectively in four consecutive time phases from accident to discharge from hospital: A) Pre-hospital phase, B) Emergency room and initial surgery, C) Intensive care unit and D) Discharge. The documentation included detailed information on demographics, injury pattern, comorbidities, pre- and in-hospital management, course on intensive care unit, relevant laboratory findings including data on transfusion and outcome of each individual. The inclusion criterion was admission to hospital via emergency room and subsequent admission to an ICU/ICM care unit or reaching the hospital with vital functions and die before admission to ICU. There were two sets of data: The standard dataset required to document the complete set of data as defined by the registry. The QM dataset (quality management dataset) required a reduced number of items. It was up to the discretion of the participating hospitals which type of dataset was used. If a hospital decided for either dataset, all patients were documented by the chosen dataset. The infrastructure for documentation, data management, and data analysis was provided by AUC - Academy for Trauma Surgery (AUC - Akademie der Unfallchirurgie GmbH), a company affiliated to the German Trauma Society. The scientific leadership was provided by the Committee on Emergency Medicine, Intensive Care and Trauma Management (Sektion NIS) of the German Trauma Society. The participating hospitals submitted their data into a central database via a web-based application. Scientific data analysis was approved according to a peer review procedure established by Sektion NIS. The participating hospitals were primarily located in Germany (90%), but a rising number of hospitals of other countries contributed data as well (at the moment from Austria, Belgium, China, Finland, Luxembourg, Slovenia, Switzerland, The Netherlands, and the United Arab Emirates). Currently, approximately about 35,000 cases from almost 700 hospitals were entered into the database per year.

This study was conducted according to the publication guidelines of the TraumaRegister DGU® (TR-DGU) and registered as TR-DGU Project ID 2019–015.

### Selection of patients

Basis for the analysis were patients from the years 2015 until 2017. In 2015, the data set has been revised and the two relevant parameters “Patient’s volition” and “assumed cause of death” were added to the TraumaRegister DGU®. “Assumed cause of death” is a retrospective assumption by the treating physicians and the registry has no information if an autopsy or else verified these assumptions. Only patients from German hospitals were included. All non-surviving patients initially admitted to the ICU were analysed. The target group of patients who died after initial discharge from ICU was defined as those with length of stay in hospital being more than 1 day longer than the cumulated length of stay on ICU. We could not discriminate patients being less than 24 h outside the ICU as documentation was performed on a daily basis.

The TR-DGU only collects the total number of days in hospital and on ICU; re-admission to ICU is not coded. Therefore, a discrimination between patients that died after readmission to ICU and patients that died on normal ward was not possible.

### Analysis

Variables were analysed using descriptive statistics (percentages and frequencies) and central tendency measures for metric variables, for continuous variables mean with standard deviation and median with interquartile range (IQR) for non-continous variables [18]. For selected findings, a 95% confidence interval (CI95) was calculated. SPSS version 22 (IBM Inc., Armonk, NY, USA) was used for the statistical analysis. The study was approved by the Ethics Committee of the Ruhr-University Bochum, Germany.

## Results

**Fig. 1:**
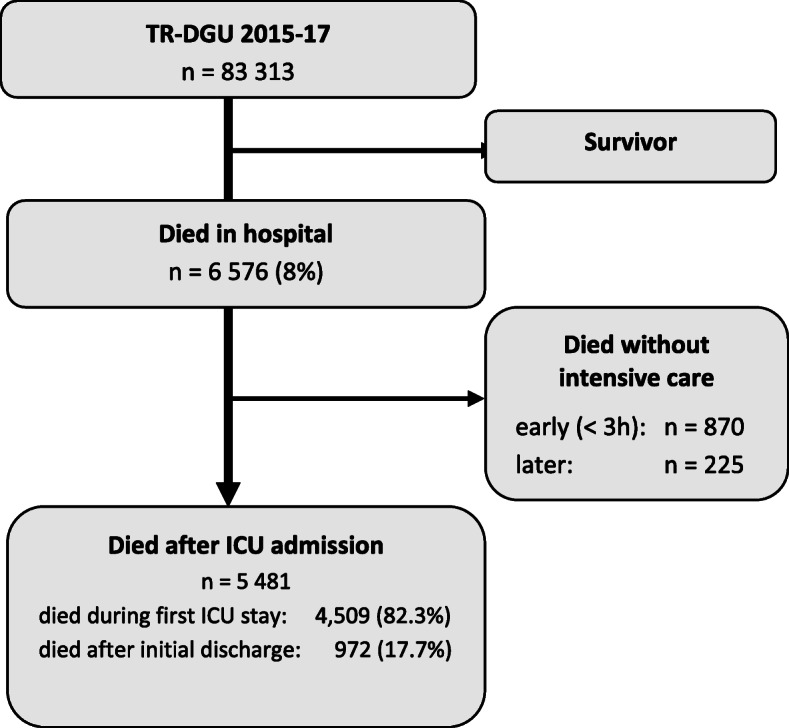
.

The selection process of the study patients is shown in Fig. 1. The excluded surviving patients had a mean age of 50.3 years, a mean ISS of 16.3, mean LOS on ICU was 6.3 days, and mean LOS in hospital was 16.9 days. Overall, 5481 patients were admitted to the ICU alive and died later in hospital. In total 972 (17.1%; CI95: 16.7–18.7) of these patients were discharged from the ICU to normal ward for some days. Differences in demographics, injury pattern, therapy and course in the intensive care unit between patients who died in ICU or later after initial discharge are shown in Table 1. Patients that died before ICU discharge were younger (mean age 68 vs. 77 years), more severely injured (mean ISS 30.0 ± 15 vs. 23.1 ± 12; RISC II prognosis for mortality 52.1 vs. 32.4%); length of stay in ICU was shorter (mean days 6.0 ± 9.4 vs. 10.0 ± 13.4), while living will limiting life-sustaining therapy was documented fewer (46.1 vs. 59.9%).

**Table 1:** Demographics, injury pattern, treatment and outcome of non-surviving patients initially admitted to an ICU

	Died during first ICU stay	Died after initial discharge from ICU
Number of patients (n)	4509	972
Male (%)	63.6	60.4
**Age**		
Age (years)	68.0 ± 20.9	76.8 ± 15.4
Aged 18–59 years (%)	25.7	11.7
Aged 60–69 years (%)	12.6	9.2
Aged 70–79 years (%)	23.7	24.8
Aged 80+ years (%)	38.0	54.4
Pre-injury ASA 3 or 4 (%)	45.1	60.1
**Injury pattern**		
Blunt trauma (%)	96.1	97.3
ISS (points)	30.0 ± 15.0	23.1 ± 12.6
Traffic accident (%)	31.4	22.3
Low fall (< 3 m) (%)	46.7	62.3
Isolated head injury AIS ≥3 (%)	32.9	34.1
No head injury (AIS ≤ 2) (%)	16.9	24.8
Combined head and other trauma (%)	50.1	41.2
AIS head > = 3 (%)	77.6	65.8
AIS chest > = 3 (%)	39.5	30.3
AIS abdomen> = 3 (%)	10.7	6.8
AIS extremities > = 3 (%)	19.3	19.3
RISC II prognosis for mortality (%)	52.1	32.4
**In-hospital course**		
Systolic blood pressure on admission < 90 mmHg (%)	21.6	8.6
Glasgow Coma Scale 3–8 on admission (%)	64.5	31.2
Received at least 1 PRBC transfusion (%)	19.1	8.5
Days in hospital	2 [1–7]	12 [6–20]
Days in ICU	2 [1–7]	5 [2–13]
Days ventilated	2 [1–5]	1 [0–7]
Non-operative treatment (%)	50.5	48.8
Sepsis (%)	13.7	19.5
Organ failure (%)	90.3	75.9
Multiple organ failure (%)	66.9	50.8
Thrombo-embolic events (%)	6.1	10.4
**Outcome**		
Living will limiting life-sustaining therapy (%)	46.1	59.9
Died within 30 days	97.2	85.0
**Assumed cause of death**		
Head injury (%)	58.2	42.6
Bleeding (%)	5.7	1.6
Organ failure (%)	25.3	29.9
Others (%)	8.8	23.4

**Fig. 2:**
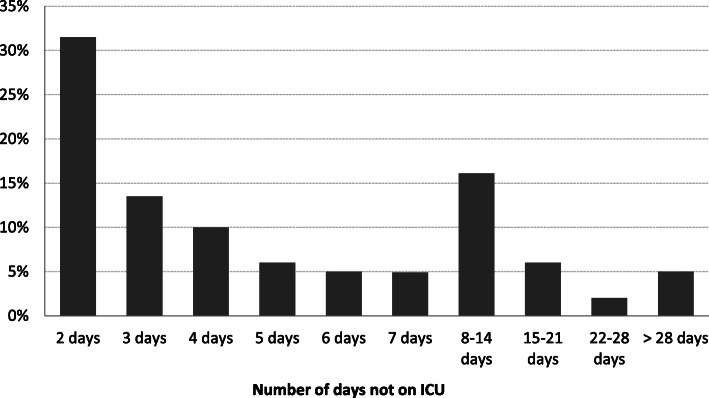
.

Continuous variables are presented as mean ± standard deviation; non-continuous variables are presented as median [interquartile range]. Abbreviations: ASA: American Society of Anastesiologists classification; ISS: Injury Severity Score; AIS: Abbreviated Injury Score; RISC II: Revised Injury Severity Score II; PRBC: packed red blood cells;

Of all non-surviving patients that were initially discharged from ICU, 31.5% were treated less than 3 days and 70.9% less than 8 days outside the ICU (Fig. 2). In addition to patient-related factors, we also analysed a potential difference with respect to the level of care of the trauma center (Table 2). The rate of patients dying within first ICU stay was the highest in level 1 trauma centers (84.7%; CI95: 83.6–85.9) and was the lowest in level 3 trauma centers (68.7%; CI95: 62.9–74.4).

**Table 2:** Non-surviving patients after initial ICU admission depending on Level of trauma center

Level of care	died during first ICU stay	died after discharge from ICU
Local trauma center (level 3)	173 (68.7%)	79 (31.3%)
Regional trauma center (level 2)	1009 (77.4%)	294 (22.6%)
Supra-regional trauma center (level 1)	3327 (84.7%)	599 (15.3%)
All patients	4509 (82.3%)	972 (17.7%)

## Discussion

The majority of in-hospital deaths after major trauma occurred during the initial ICU treatment period. This study showed that a relevant group (17.1%) of all non-surviving patients initially admitted to the ICU were discharged from ICU at least once and treated outside the ICU for more than 1 day.

Multiple studies focussed on in-hospital mortality after discharge from the ICU [19–23] Pearse et al. performed a prospective observational study on mortality after surgery in Europe [9] In emergency surgery, they found a mortality rate of 11.1% of all patients initially discharged alive from ICU to normal ward. They concluded that ICU resources were not allocated to patients at greatest risk of death. A variety of factors influencing readmission rate have been identified. Several studies showed a relationship between daytime and weekday of discharge from ICU and outcome. They confirmed that unplanned discharges from the ICU during night-time and on weekends were independent risk factors for readmission to the ICU and increased mortality [19, 24, 25]. Others confirmed older age, severity of illness on the day of admission to the ICU and day of discharge from the ICU as well as comorbidities as independent risk factors [8, 26–28]. We did not compare surviving and non-surviving patients after ICU-discharge but non-survivors with or without initial discharge from ICU. Non-survivors after initial ICU discharge were older, less severely injured and the severity of comorbidities as measured by the American Association of Anaesthesiologists Classification (ASA) [29] were higher. Interestingly, the injury pattern and the course in the ICU differed in several points: While the percentage of blunt trauma and isolated head injury did not differ relevantly, the proportion of trauma patients without head injury was much higher in non-survivors discharged from the ICU. Percentage of patients after low fall was higher in non-survivors discharged from ICU. Regarding the ICU treatment period one hypothesis could be that patients dying after ICU discharge underwent less intensive care as they were less severely injured. They may have been underestimated by the treating physicians and were discharged from the ICU prematurely. However, the mean length of stay on the ICU long (mean days on ICU: 4) as well as the days with ventilatory support (mean days ventilated: 5.8). Low fall is a known surrogate marker for frailty, predicting long-term mortality in older trauma patients [30].

In general, fatalities after the initial discharge from the ICU may be explained by either a limitation of life-sustaining intensive care treatment with palliative care outside the ICU due to a living will or by patients who were discharged from the ICU after initial recovery but suffered a late fatal complication.

The causes of mortality of these patients remained unclear. A documented living will to limit sustaining therapy was present in 60% of patients who died after discharge from the ICU. However, we couldn’t state that all of these patients actually underwent palliative care and were discharged from the ICU due to therapy limitation. A living will was also present in 46% of non-survivors who died during their initial ICU stay. Consequently, a living will to limit life-sustaining treatment did not per se lead to an ICU discharge in this setting. The ICU stay was also rather long so that we cannot assume that there was a significant number of short (e.g. 24 h) ICU try-outs for the very old critically ill patients with consecutive withdrawal of intensive care treatment in those patients with failing recovery.

After initial recovery late complications like sepsis (19.5%) and thromboembolic complications (10.4%) resulted in death of the patient. Assumed causes of death differed to typical reasons for early mortality, i.e. bleeding, head injury and organ failure in 23.4% of cases. We can only assume which other causes of death occurred as the registry data gives no more detailed information regarding the parameter “assumed cause of death”. However it appears logical that all late deaths after ICU discharge were due to a potentially occult complication.

Surprisingly, we found relevant differences between trauma centers of different care levels. About 5% of our study population was treated in a local level 3 trauma center, 24% in a regional level 2 trauma center, and the vast majority in a supra-regional or level 1 trauma center. The percentage of non-survivors after initial ICU discharge was highest in level 3 centers and lowest in level 1 centers (Table 2). Although it seems intuitive that fewer late fatal complications occur in experienced trauma centers, previous studies focusing on the relationship between center volume and complication rates were inconclusive. Several studies found a correlation between high volume and improved outcome, especially in high-risk surgical and trauma patients [31–33] However, other studies found no association between institution or surgeon volume and survival [34–36]. Bell et al. [37] investigated the relationship between trauma center volume and in-hospital outcome and showed that higher hospital volumes were associated with decreased likelihood of mortality but not for complication or FTR.

We cannot conclude from our data that our findings were related to differences in availability of ICU beds, other recourses or higher rates of failure to rescue (FTR). Different institutions may have different limitations in resources and, for example, comfort care in the high-dependency unit in one hospital may be equivalent to staying in the ICU for comfort care in another hospital.

Although our study identified a cohort of FTR patients, we could not measure and compare FTR rates. Firstly, we did not know whether patients that died during their initial stay in the ICU suffered from complications or what the influence on mortality might have been. Secondly, we did not know complication rates of surviving patients to show the rate of survived complications.

FTR rates are considered as a quality indicator superior to complication or mortality rates as they are more associated with institutional factors [38–41]. The rate was based on elective surgical populations and reclassified deaths not caused by adverse events. Holena et al. [38] demonstrated that this approach lacks validity in trauma because patients often die as a direct consequence of injury without any adverse events. They also argued that another common approach to simply exclude deaths without recorded adverse events reduces the reliability of the FTR rate. They proposed to include all deaths but excluding those with an expected mortality > 50%. Though the limit is still under discussion and yet to set definitely.

Moore et al. [39] performed a systematic review on complication rates as a trauma care performance indicator. Only three complications (pneumonia, pulmonary embolism and deep vein thrombosis) were recommended to evaluate acute trauma care hospitals. A recent study by Chung et al. [40] showed that 33% of all major complications in trauma patients occurred on normal surgical wards.

Rauf et al. [42] observed that the time distribution of in-hospital mortality in severely injured patients shows a constant decrease. About 61% of all deaths occur within the first 48 h after admission. Mean time to death was about 6.5 days with a median of 2 days.

This might suggest to focus on the initial hours and days after trauma in terms of quality improvement. However, we believe that our study identified a relevant field of potential improvement to decrease mortality rates even further.

Therefore we propose the mortality rate of patients after initial discharge from ICU as a reliable quality indicator for trauma care. Its calculation and extraction from registries is simple and independent from definition and measurement of adverse events.

### Limitations

This study has several limitations: It is a retrospective study of a registry and therefore there is potential for bias or residual confounding from factors we did not measure. We only have information on acute care hospital outcome without any follow-up data on discharged patients. Furthermore, we did not discriminate between patients that died on the normal ward on those readmitted to ICU. We also cannot give information on patients treated less than 2 days outside the ICU. Our study protocol included these patients in the group of patients that died within first ICU course. We cannot give detailed information whether and how a living will to limit life-sustaining therapy contributed to the observed mortality rate. The registry does not discriminate between “the patient did not want any more intensive care medicine though intensivists recommended” and “therapy was limited due to grave prognosis”. Furthermore, there are patients who are discharged from ICU (“terminal discharge”) due to poor prognosis, and patients who die before this terminal discharge can be planned. We included patients from Germany, as differences in health systems, i.e. limited ICU resources or cultural differences i.e. handling of living will statements might have influenced our results.

However, the strength of this study is the large study population with detailed patient characteristics representing most severely injured patient in one representative developed country and health system.

## Conclusion

17.7% of all non-surviving patients after severe injury initially treated on an ICU, have been discharged to the general ward prior to their death. These deaths can only partially be explained by a limitation of life-sustaining therapy due to a living will. They might represent an important group of patients that might have died from potentially avoidable and treatable complications. The incidence of mortality after discharge from the ICU might be a valuable quality indicator for trauma care of the severely injured patients provided that patients undergoing palliative care are excluded reliably. Further studies are required to identify patients at risk of dying after discharge from the ICU at an early stage and characteristics responsible for the adverse outcome.

## Data Availability

The datasets used and analyzed during the current study are available from the corresponding author on reasonable request.

## Abbreviations

AIS: Abbreviated injury score
ASA: American Society of Anaesthesiologists classification
ED: Emergency department
FTR: Failure to rescue
ICU: Intensive care unit
ISS: Injury severity score
PRBC: Packed red blood cells
TR-DGU: Trauma registry DGU (German Trauma Society)
